# High resolution melting analysis and detection of *Leishmania* resistance: the role of multi drug resistance 1 gene

**DOI:** 10.1186/s41021-021-00210-5

**Published:** 2021-08-11

**Authors:** Maryam Fekri Soofi Abadi, Alireza Moradabadi, Reza Vahidi, Saeedeh Shojaeepour, Sara Rostami, Iman Rad, Shahriar Dabiri

**Affiliations:** 1 Pathology and Stem Cell Research Center, Department of Pathology, Afzalipour Medical School, Kerman University of Medical Sciences, 22 Bahman Blvd, Kerman, Iran; 2 Department of medical laboratory science, Khomein university of medical science, Khomein, Iran; 3 Latner Thoracic Surgery Research Laboratories, Division of Thoracic Surgery, University Health Network, Toronto, Ontario Canada

**Keywords:** Unresponsive leishmaniasis, HRM method, Pentavalent antimonial compounds, Multi-drug resistance 1 gene

## Abstract

**Background:**

Pentavalent antimonial compounds are currently used to treat leishmaniasis and resistance to these drugs is a serious problem. Multidrug resistance protein is an efflux pump of the cell membrane that expels foreign compounds. This study designed to evaluate the mutations in the multi-drug resistance 1 (MDR1) gene, in biopsy specimens of *Leishmania tropica*, with high resolution melting (HRM) method. In this experimental study, genomic DNA was extracted from 130 patients with skin leishmaniasis. Then, nucleotide changes were investigated throughout the gene using HRM and sequencing methods. The samples categorized in 5 groups by differences in the melting temperature (Tm).

**Result:**

The nucleotide changes analysis showed that 61% of the samples of different groups that were unresponsive to drug had mutations in the MDR1 gene, which were also confirmed by the sequencing method. These mutations can be one of the factors responsible for non-responsiveness to the treatment.

**Conclusion:**

According to the findings, it seems that mutation in MDR1 gene could be responsible for drug resistance to pentavalent antimonial compounds. Furthermore, HRM method can be used to diagnose drug resistance in leishmaniasis. It is also recommended that further studies be done regarding the importance of drug resistance in the *leishmania* affected patients.

## Introduction

Cutaneous leishmaniasis is one of the major diseases in tropical and subtropical areas [1] which is caused by parasites of *Leishmania tropica* and *Leishmania major* [2–5]. It is a health problem in 88 countries and its controlling and preventing is one of the top priorities of the international community, including the World Health Organization [6–8]. Currently, pentavalent antimony compounds, including glucantime and sodium stibogluconate (SSG), are used to treat leishmaniasis and through various direct and indirect mechanisms may eliminate this parasite. Besides inhibition of the activity of enzymes involving in the fatty acid oxidation pathway [9, 10] and stimulation of macrophages to produce lethal molecules such as nitric oxide (NO) and reactive oxygen species (ROS) [11], these compounds via creating the flow of intracellular thiol (decrease of parasitic thiol), the disturbance in the oxidation potential and by inhibiting trypanothione reductase lead to oxidative stress-dependent parasite death [12].

Studies on the failure of a pentavalent antimony treatment have recently been reported in several countries [9]. Since uncovering the molecular mechanisms involved in drug resistance will help to treat patients, efforts to identify these mechanisms are ongoing.

Recent our experiment evidenced that expression of multidrug resistance protein 1 (MDR1) or CD243 is different in various forms of cutaneous leishmaniasis [13]. This ATP-dependent drug efflux protein pumps several substances out of cells and can reduce drug accumulation in resistant cells [14]. About 65% of the Mediterranean *leishmania* strains have amplified MDR1 genes [12]. In addition, it has been made clear that overexpression of this gene has been associated with an increased resistance of *leishmania* to miltefosine [15]. Overall, the mutation of this gene appears to play a key role in the phenomenon of drug resistance [16–18]. Therefore, screening of patients with this mutation has an important role in the treatment of patients with leishmaniasis. Due to the limitations of gold standard method (sequencing), trying to identify simple, feasible, and high-performance methods is inevitable. One of these methods is high resolution melting analysis (HRM). This new, accurate, and affordable method is a post-PCR analysis of genetic variation that performed in a sealed tube [19, 20].

Given the importance of MDR1 gene mutations in the drug resistance of *leishmania* and the lack of similar study, in the present experiment, we evaluated the capacity of HRM assay for detection of MDR1 gene mutation in genomic DNA of tissue specimens of patients with skin leishmaniasis.

## Materials and methods

### Sample collection

Tissue specimens from 130 patients (chemoresistant samples: 61% (80 samples) and chemosensitive samples: 39% (50 samples)) with cutaneous leishmaniasis along with one standard sample (sensitive strain of *leishmania tropica*; MHOM/IR/10/175) were obtained from Dermatology Department of Afzalipour Hospital in Kerman, Iran.

### DNA extraction

Genomic DNA was isolated by using QIAamp DNA Mini Kit (QIAGEN, Germany). We used the manufacturer’s protocol for extraction. DNA concentration was determined by Nano Drop® at a wavelength of 260 nm. Also, the absorption ratio of a pure DNA at 260 and 280 nm was calculated (A_260/280_).

### HRM analysis

Specific primers were designed to amplify the MDR1 encoded region of the MDR gene. For this purpose, we aligned the sequence of HM854717.1 through HM854725.1 (partial sequence) with the whole sequence of the MDR1 (U63320.1). The forward primer sequence was selected from the nucleotides before the conserved points of the 10 mentioned sequences (HM854717.1-HM854725.1 and whole sequence of the MDR1). The reverse primer is selected among sequences that located after the conserved points (160 nucleotides after the forward primer). The designed primers had the following sequence and were synthesized by TibMolbiol (Berlin, Germany):

MDR1-F: 5′-ATTGTCGCTTCTGGGGTTG-3′.

MDR1-R: 5′-ATCGTGTCGCTTGTGTCAC-3′.

Real-time PCR was performed using 10 μL of Type-it Master Mix (QIAGEN, Germany), 2 μL of DNA, 0.7 μL of each of forward and reverse primers (10 pmol) and 6.6 μL of nuclease-free water in a total volume of 20 μL. Thermal cycling conditions included an initial activation step at 95 °C for 5 min followed by 40 cycles including a denaturation step at 94 °C for 20 s and a combined annealing/elongation step at 60 °C for 30 s. The reaction took place in rotor gene 6000 (QIAGEN, Germany). For HRM analysis, the PCR products were melted by warming up the temperature from 40 °C to 95 °C (0.007 °C.s^− 1^) and 20 fluorescence acquisitions were recorded in each temperature. The normalized graph and the normalized temperature-shifted difference graph (difference graph) from the gene scanning analysis were used to analyze the data. HRM data were analyzed using rotor gene 6000 (QIAGEN, Germany) software. Based on the melting temperature (Tm), studied samples were categorized in five groups. Namely, group 1 was the standard and treatment-sensitive samples and each of the other groups also illustrated samples with similar melting temperature.

### Agarose gel electrophoresis

After amplification, the amplicon was electrophoresis in agarose gel. The gel (1.5%) was prepared in Tris/Borate/EDTA (TBE) buffer and intercalating dye (SYBR Safe) added to gel solution to make the DNA visible under UV. After casting the gel, creating wells, and sinking the gel in TBE buffer, PCR products were loaded into wells. The DNA ladder was used to determine the sizes of sample bands.

### Sequencing

To confirm nucleotide changes and compare the nucleotide differences among samples of different groups, some of the samples (10% of each group) were amplified by MDR1-R and MDR1-F primers and bilateral sequencing was accomplished by Bioneer Company in South Korea. The results of sequencing were studied by the choromas pro.v.2.1 software and sequence alignment was carried out. Alignment diagram was drawn by the use of Vector NTI advance 11 software.

## Results

### Amplification and electrophoresis

**Fig. 1:**
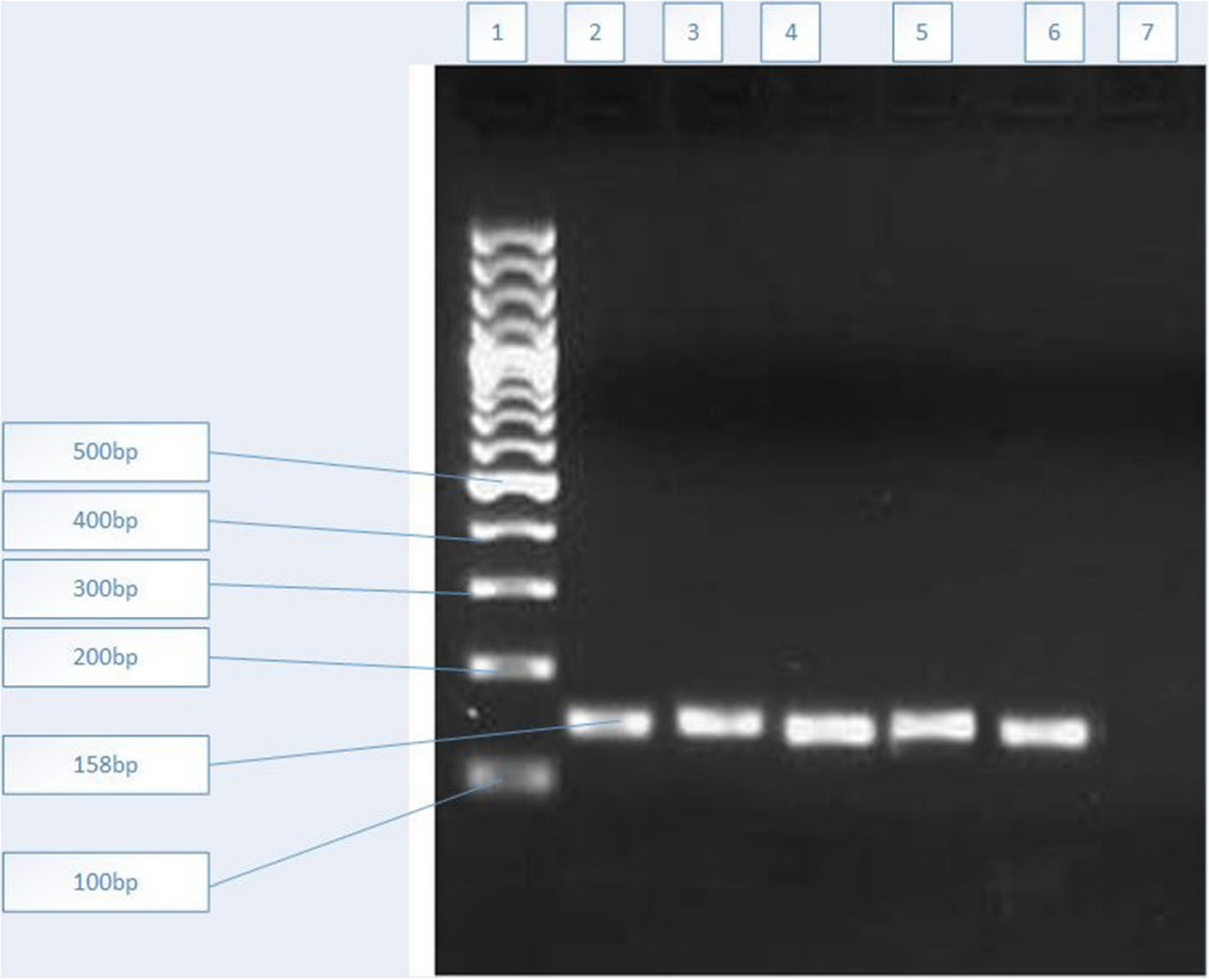
Electrophoresis of the PCR product in the samples on the 1.5% agarose gel; from left to right: The leftmost lane depicts a molecular marker (100 bp) and lanes 2, 3, 4, 5 and 6 indicate amplified fragment (158 bp) by MDR1 primers in 5 samples. Lane 7 is non template control (NTC)

The amplification was confirmed by conventional PCR and gel electrophoresis. As illustrated in Fig. 1, all samples had 158 bp amplification products.

### Real-time PCR and HRM results

**Fig. 2:**
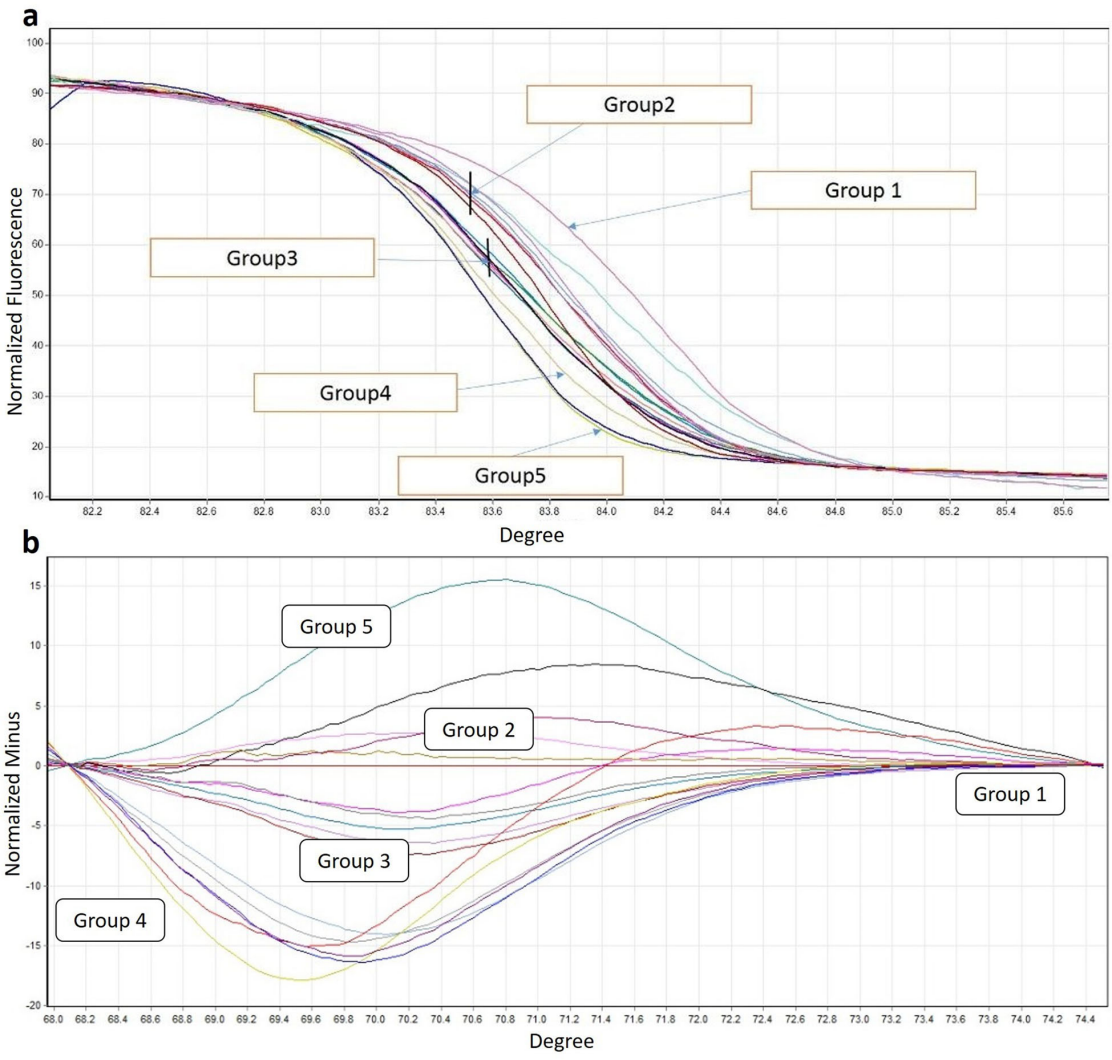
HRM-based categorization of samples into 5 groups. The group 1 gave the highest melting temperature and the group 5 had the lowest melting temperature (**a**). Each group had at least 0.3 °C difference to other groups. Group 1 actually refers to standard sample and samples of treatment-sensitive patients. Each of the other groups also illustrates samples with similar melting temperature. Difference plot was presented in (**b**)

Corresponding primers amplified a 158 bp product. In HRM analysis, all samples had melting peaks with certain Tm. Different Tm can be attributed to genomic differences in the studied area. To further differentiate, a high-resolution difference curve was plotted by subtracting the melting curve of each sample from the baseline (reference) curve. Accordingly, samples with similar curves were placed in separate groups (Fig. 2).

### Alignment

**Fig. 3:**
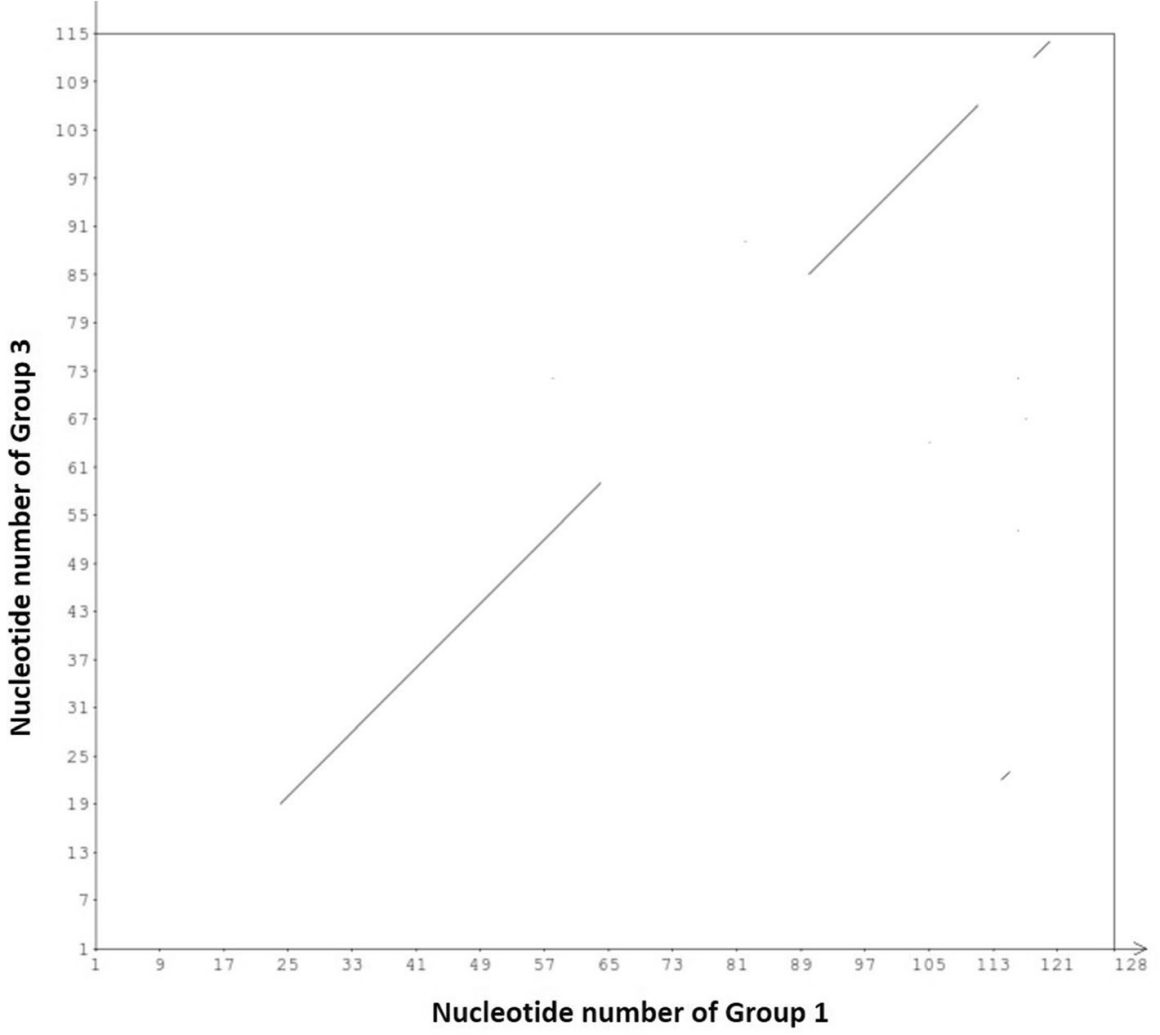
The alignment chart of groups 1 and 3. The breaks illustrated the nucleotide differences in the MDR1 region. This is an example of the alignment between groups

**Fig. 4:**
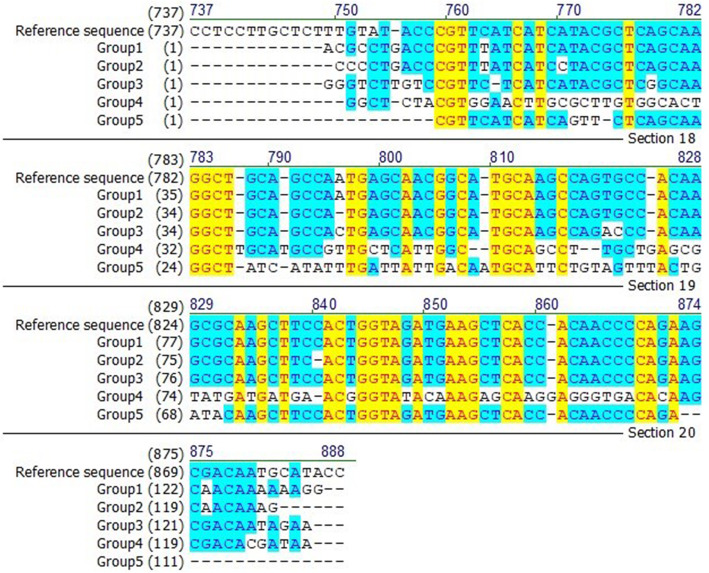
The alignment of the reference sequence (U63320.1) with nucleotide sequences of the different groups. As is clear, the identical, conservative, and non-similar nucleotides were illustrated by yellow, light blue, and white background and by red, dark blue, and black foreground, respectively. As evidenced, some insertion and mutation were occurred in the MDR1 gene of groups

To compare the amplified products, some of the samples were sent for sequencing. Next, the nucleotide sequence of different groups was subjected to alignment with reference sequence. In this method, each nucleotide of a sample is compared with all nucleotide of the reference sample and any similarity is shown by a dark dot. Since the highest difference was obtained between groups 1 and 3, so, the Fig. 3 is representing their corresponding alignment chart. As mentioned before, different melting temperature can be attributed to genomic differences in the studied area. As presented in Fig. 4, some insertion and mutation were detected in the sequence of MDR1 gene of most of the patients that we studied.

## Discussion

Currently, chemotherapy using the pentavalent antimonials (such as glucantime) is applied more than other methods in the treatment of leishmaniasis. However, the use of glucantime in patients with cutaneous leishmaniasis has some side effects on vital organs such as kidney, liver, heart, and blood [18, 21–24]. Studies conducted in different parts of the world showed drug resistance to glucantime in some people with leishmaniasis [24–26] and had caused a lot of concern after about 65 years of usage. Although the resistance mechanisms are poorly understood, different factors such as genetic factors, proteinaceous factors, enzymatic factors, and alteration of drug uptake and accumulation appear to be effective in causing this condition [17, 18, 25–29].

ATP-binding cassette (ABC) transporters such as P-glycoprotein (P-gp) are transmembrane proteins, which is encoded by the MDR1 gene. P*-*gp is an ATP*-*dependent drug efflux pump which can reduce the drug’s accumulation in resistant cells and often increases the drug resistance [30–32]. The MDR1 gene of *leishmania* is 37% similar to human MDR gene and located on chromosome 34. Heretofore, the association of increased resistance to several drugs with overexpression of this gene has been proven [14, 33–35]. In line with these studies, our experiment in 2019 showed that the expression of this gene is different in various forms of the disease (acute, chronic lupoid, and chronic non-lupoid) and can be considered as an important factor in the drug resistance of *leishmania* parasite [5].

Although sequencing is considered as a gold standard approach for detection of mutations, the major obstacles are overpriced instrumentation, inaccessibility, and need to striking technical expertise. Therefore, and according to the importance of drug resistance, modern alternative techniques can provide an opportunity for *leishmania* control and treatment. Flow cytometry, RFLP-PCR, and HRM are among these techniques. For instance, Alizadeh and colleagues by using RFLP-PCR method illustrated that about 11% of the collected samples of cutaneous leishmaniasis from some endemic areas of Iran (Yazd, Mashhad, and Kashan provinces) had mutations in MDR1 gene [36]. Tsirigotakis et al. also evaluated drug resistance in *leishmania* isolates using flow cytometry [37]. Since Rhodamine-123 is an established substrate for P-gp 170 and its efflux rate is largely dependent on the number of efflux pumps, they introduced the efflux rate of this dye as an indicator of drug resistance.

Previously, HRM assay with direct sequencing confirmation possibility was reported as a rapid and inexpensive method for detection of mutations in pathological [38]. Furthermore, this test can be used as a pre-screening method to decide whether sequencing should be done on a sample. To discriminate between wild-type and mutant DNA, the genomic DNA is amplified with specific primers and in the presence of a fluorescent intercalating dye. Then, the fluorescent melting curve of amplicons is analyzed [20]. Regarding validation, Heideman and co-workers challenged the performance of HRM assay for detection of mutations in genomic DNAs of 68 non-small cell lung cancer specimens [38]. They evidenced that this assay had a sensitivity of ≤5% of mutated DNA in a background of wild-type DNA and could be applied for screening of mutations.

In the present experiment, we evaluated the capacity of HRM assay for detection of MDR1 gene mutation in genomic DNA of tissue specimens of 130 patients with skin leishmaniasis. Subsequent sequencing of the HRM products was applied to confirm the presence of the nucleotide alteration.

Figure 1 illustrated that MDR1 gene was amplified in our samples. Based on the Tm, studied samples were categorized in five groups. Group 1 actually refers to standard sample and samples of treatment-sensitive patients. Each of the other groups also illustrates samples with similar melting temperature (Fig. 2). Our results showed that 61% of the specimens had mutations in the MDR1 gene region. To confirm findings and compare the amplified products, sequencing and then alignment analysis was applied (Figs. 3 and 4). The analysis showed that the type of mutations in each group is similar but different from the mutations in the other groups. Interestingly, no discrepancy was found between HRM and sequencing findings as well as triplicate analysis confirmed the reproducibility of HRM assay.

## Conclusions

Totally, the present experiment established that HRM assay is sensitive and suitable enough to screen mutations of MDR1 gene. Furthermore, rapidity, simplicity, feasibility, and cost-effectiveness are other advantages that support HRM use to detect mutations. In addition, unlike methods such as RFLP-PCR, HRM assay detects mutations anywhere in the amplicon and does not require knowing the exact location of the mutation. Nevertheless, this assay could not identify the specific nucleotide change. It should be noted that this inability is predictable for a screening test. As a suggestion, additional experiments for more validation of HRM assay, as a screening test for detection of resistant patients, as well as determination of the prevalence of genetic factors affecting the resistance of antimony compounds in endemic areas of cutaneous leishmaniasis are necessary.

### Limitations

The present study has potential limitations. The lack of information about the duration, amount, and type of drugs which were used to treat the studied patients can be considered as the most important ones. It should be noted that due to the overlap of melting curves, identifying single-point mutations whose melting temperatures do not differ much can be challenging.

## Data Availability

Please contact corresponding author (S.D) for data requests.

## Abbreviations

MDR1: Multi-drug resistance 1
HRM: High resolution melting
Tm: Melting temperature
SSG: Sodium stibogluconate
NO: Nitric oxide
ROS: Reactive oxygen species
PCR: Polymerase chain reaction
NTC: Non-template control
ABC: ATP-binding cassette
P-gp: P-glycoprotein
TBE: Tris/Borate/EDTA
